# Shear bioreactors stimulating chondrocyte regeneration, a systematic review

**DOI:** 10.1186/s41232-019-0105-1

**Published:** 2019-08-08

**Authors:** Negar sharifi, Anneh Mohammad Gharravi

**Affiliations:** 1 Student Research Committee, Shahroud University of Medical Sciences, Shahroud, Iran; 2 Tissue Engineering and Stem Cells Research Center, Shahroud University of Medical Sciences, Shahroud, Iran

**Keywords:** Shear, Bioreactors, Chondrocyte, Regeneration

## Abstract

It is commonly accepted that the mechanical stimuli are important factors in the maintenance of normal structure and function of the articular cartilage. Despite extensive efforts, the cellular mechanisms underlying the responses of articular chondrocytes to mechanical stresses are not well understood. In the present review, different types of shear bioreactor and potential mechanisms that mediate and regulate the effect of shear on chondrocyte are discussed.

For this review, the search of the literature was done in the PubMed, Scopus, Web of sciences databases to identify papers reporting data about shear on chondrocyte. Keywords “shear, chondrocyte, cartilage, bioreactor” were used. Studies published until the first of March 2018 were considered in this paper. The review focused on the experimental studies conducted the effect of shear stress on cartilage tissue in vivo and in vitro. In this review, both experimental studies referring to human and animal tissues were taken into account. The following articles were excluded: reviews, meta-analysis, duplicate records, letters, and papers that did not add significant information. Mechanism of shear stress on chondrocyte, briefly can be hypothesized as (1) altered expression of aggrecan and collagen type II, (2) altered cartilage oligomeric matrix protein (COMP) serum levels, consequently, organizing the arrangement binding of glycosaminoglycans, integrins, and collagen, (3) induction of apoptosis signals, (4) altered expression of integrin.

## Background

It is now commonly accepted that the mechanical stimuli are important factors in the maintenance of normal structure and function of the articular cartilage and changes its morphology in response to mechanical stimuli. Despite extensive efforts, the cellular mechanisms underlying the responses of articular chondrocytes to mechanical stresses are not well understood [1].

The mechanisms by which chondrocytes actively respond to mechanical stimuli are important for understanding the modulators and signaling pathways involved in the pathogenesis of major disabling diseases, such as rheumatoid arthritis (RA) and osteoarthritis (OA) [2]. But, due to the complexity of the signaling mechanisms, the detailed pathways remain unclear.

Different mechanical stimuli such as compressive and tensile forces modulate chondrocyte function. Articular cartilage is the highly specialized hydrated (80% water) connective tissue that experiences the solute transport in the cartilage and movement of fluid during the loading and unloading conditions (exploding water during loading, and draw back into the tissue during unloading) [3].

By the movement of fluid within cartilage, chondrocytes experience potential fluid shear stress that affects chondrocytes proliferation, apoptosis, growth and differentiation, and extracellular matrix production [4]. A number of pathways involved in transduction of the mechanical stimuli of shear stress to intracellular signaling, but despite the extensive effort, exact mechanisms remain unclear. Thus, in the present review, different types of shear bioreactor and potential mechanisms that mediate and regulate chondrocyte proliferation and matrix production are discussed.

## Main text

**Fig 1.**
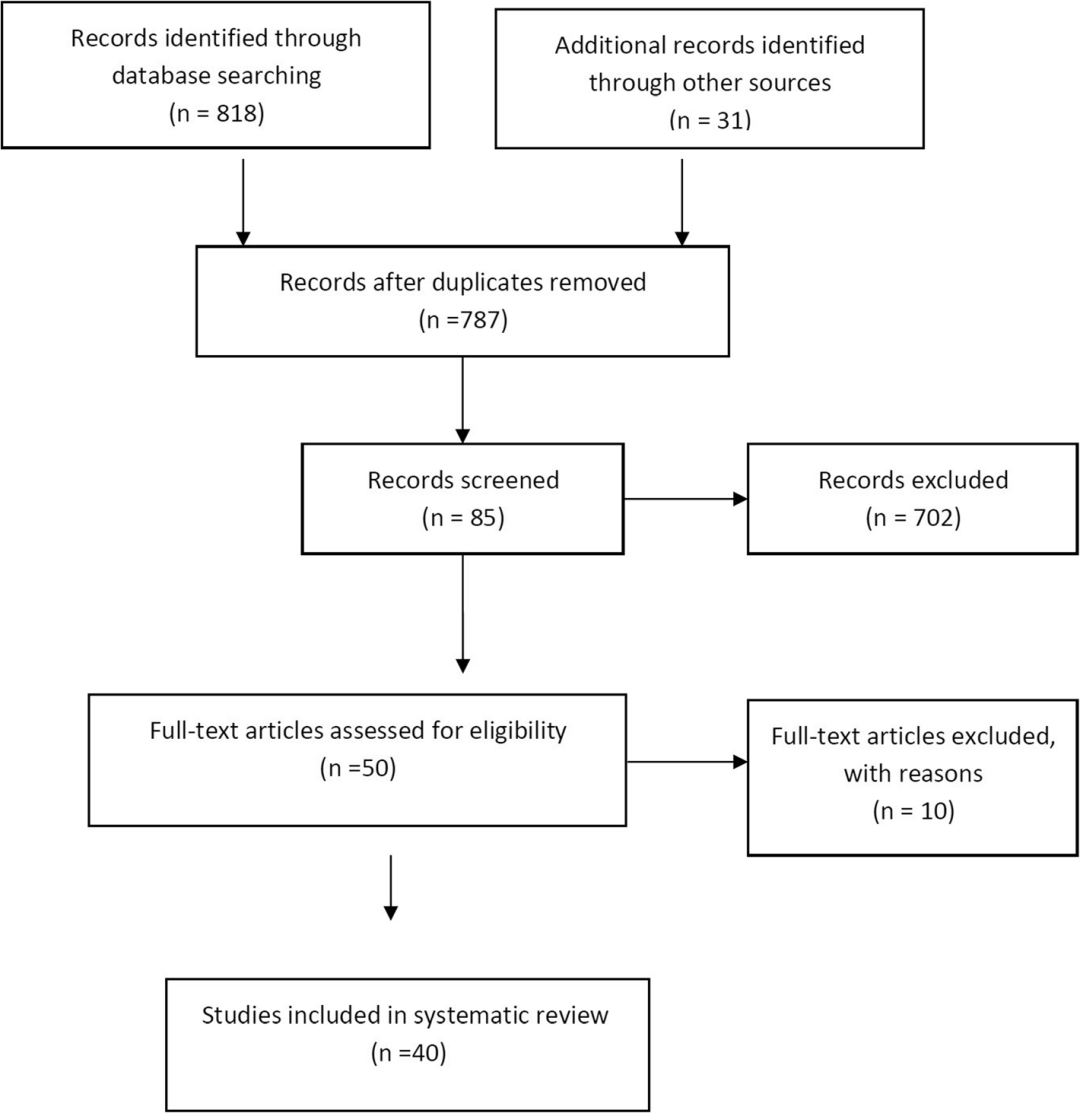
Flow chart illustrating the number of investigations and studies included in the analysis

For this review, the search of the literature was done in the PubMed, Scopus, Web of Sciences databases to identify papers reporting data about the effect of shear stress on chondrocyte. Keywords “shear, chondrocyte, cartilage, bioreactor” were used. Studies published until the first of March 2018 were considered in this paper. The review focused on the experimental studies conducted shear stress on cartilage tissue in vivo and in vitro. In this review, both experimental studies referring to human and animal tissues were taken into account. The following articles were excluded: reviews, meta-analysis, duplicate records, letters, and papers that did not add significant information (Fig. 1). Data assessment was conducted independently by 2–6 investigators using predefined terms.

### Cartilage

Articular cartilage as a highly specialized avascular, aneural, and alymphatic, connective tissue is composed largely of water, collagen, proteoglycans, and cells. The primary function of articular cartilage is to provide a smooth well-lubricated surface for synovial joint and to facilitate the transmission of loads. The composition and structure of articular cartilage have a direct role in its function as a lubricious, load-bearing tissue. To achieve a deep understanding of load-bearing properties, two major sets macromolecules, the proteoglycans, and collagens must first be well understood since structural interactions between these macromolecules resist compressive loads and retain water [5] (Tables 1, 2, 3, and 4).

**Table 1:** Biochemical composition of hyaline articular cartilage [6, 7]

	Water	Collagens	Proteoglycans	Other molecules
%	70–80(per ww)	50–75(per dw)	15–30(per dw)	
Property	Interstitial fluid	Collagen type II	Aggrecan, (hyaluronan + chondroitin and keratan sulfates	Fibronectin, cartilage oligomeric protein, thrombospondin, tenascin, matrix-GLA (glycine-leucine-alanine) protein, chondrocalcin, and superficial zone protein
Function	Transporting both nutrients and waste within the tissue	Fibrillar and globular collagen types, such as types V, VI, IX, and XI Intermolecular interactions as well as modulating	Comprised of a protein core with attached polysaccharide chains (glycosaminoglycans).

**Table 2:** Zonal structure of hyaline articular cartilage: from the articulating surface down to the subchondral bone [6, 7]

Zone	%	Collagen	Collagen alignment	Shape of cell	Proteoglycan	Property
The superficial (tangential)	10–20	Small diameter, densely packed collagen fibers	Parallel to the cartilage	Flattened, discoidal shapes	Low proteoglycan	Low permeability
The middle, or transitional	40–60	–	Arcade-like structure	Spherical in shape	Reaches its maximum	–
The deep zone/radial	30%	Collagen large fibers	Perpendicular to the articular surface	Columnar organization. elongated	Proteoglycan much lower than in the middle zone	“Tidemark”
The calcified zone	–	–	–	–	–	transitions into the subchondral bone

**Table 3:** Territorial Structure of hyaline articular cartilage [8]

	Location	Collagen fibers	Proteoglycans	Function
1	Pericellular matrix chondron	Type II, VI, and IX concentrated in the pericellular network of thin fibrils as fibronectin.	Mainly proteoglycans as aggrecan, hyaluronan and decorin, glycoproteins, and other non-collagenous proteins	Functional role to initiate signal transduction within cartilage with load bearing
2	(The territorial matrix) This region is thicker than the pericellular matrix	Fine collagen fibrils, forming a basketlike network around the cells Type VI collagen microfibrils but little or no fibrillar collagen.	High concentrations	May protect the cartilage cells against mechanical stresses and may contribute to the resiliency of the articular cartilage structure and its ability to withstand a substantial load
	The interterritorial matrix largest of the 3 matrix regions; it contributes most to the biomechanical properties of articular cartilage	Large collagen type IV fibers Randomly oriented bundles of large collagen fibrils, as zonal structure collagen type II, type XI collagen and type IX collagen	Are abundant	Bulk of articular cartilage permitting association with other matrix components and retention of proteoglycans. These collagens give to the cartilage form, tensile stiffness, and strength

**Table 4:** Properties of articular cartilage chondrocyte

Chondrocyte	
Role	Development, maintenance, and repair of the extracellular matrix (ECM).
Origin	Mesenchymal stem cells
Volume	2% of the total volume of articular cartilage.
Shape, number, and size	Vary in shape, number, and size, depending on the anatomical regions of the articular cartilage.
Respond to stimuli	Respond to a variety of mechanical stimuli and growth factors
Replication	Detectable cell division, limited potential for replication
Synthesis matrix	Responsible for both the synthesis and the breakdown of the cartilaginous matrix.
Differentiation	Highly differentiated cell, highly specialized, metabolically active cells
Adaption by low oxygen	Well adapted by low oxygen consumption to conditions

Zonal structure of hyaline articular cartilage from the articulating surface down to the subchondral bone is shown in Table 2. Territorial structure of hyaline articular cartilage is shown in Table 3.

### Chondrocyte

Within the cartilage matrix, the chondrocyte is the only responsible cell type for synthesis extracellular matrix and constitute about 2% of the total volume of articular cartilage.

### Effect of mechanical stimuli on chondrocyte

Interactions between chondrocytes and the ECM, consequently, homeostasis maintenance of the articular cartilage modulated by several stimuli such as mechanical stress, soluble mediators, and matrix composition. Mechanical stimuli affecting chondrocytes are divided into four categories (dynamic compression, fluid shear, tissue shear, and hydrostatic). Here, we focused on the effect of shear stress on chondrocyte metabolism. Also, four general categories of shear bioreactors are discussed [9, 10].

### Effect of shear stress on chondrocyte metabolism

As mentioned above, cartilage is a highly hydrated connective tissue. Approximately, 70% of water is expelled when the tissue is loaded in compression resulting in potential fluid shear stress at or near the cellular membrane. The water was osmotically drawn back when the tissue is unloaded [11–13]. Therefore, chondrocyte can experience fluid shear stress when water is relocated during compression [14].

Shear stress as a mechanical stimulation has been shown to affect chondrocytes through changes in membrane potential, solute transport, or cellular deformation. It is hypothesized that articular chondrocyte metabolism is modulated by direct effects of shear forces that act on the cell through mechanotransduction processes and the properties of the cross-linked type II collagen fibrils.

### Experimental studies with shear stress

Four general categories of shear bioreactors have been carried out including contact shear, fluid flow, direct fluid perfusion, and low shear “microgravity” bioreactors (Tables 5, 6, 7, and 8)

**Table 5:** Effect of experimental contact shear on chondrocyte proliferation and matrix composition

	Hz	% strain	Cell proliferation	Collagen	GAG	Proteoglycan	Scaffold
[15]	1	2	Chondrocyte	40% increase	Not measured	25% increase	(Cpp) calcium poly phosphate
[16]	0.01	0.4–1.6	Chondrocyte	40% increase	Not measured	25% increase	Cartilage disk
[17]	0.1	0.5–6	Chondrocyte	30–35% increase	Not measured	20–25% increase	Cartilage explant
[18]	0.0.1	1–3	Chondrocyte	50% increase	Not measured	25% increase	Cartilage explant
[19]	0.05–0.5	–	Chondrocyte	Not measured	Not measured	Not measured	Bovine nasal cartilage
[20]	1	–	Chondrocyte	Not measured	Not measured	Not measured	Agarose
[21]	1	2.5%	Chondrocyte	Increase	Increase	Increase	Agarose gels
[22]	0.1	3	Chondrocyte	30–100% increase	Increase	100–200% increase	Cartilage explant disks
[23]	0.5	–	Chondrocyte	Not measured	Not measured	Not measured	No scaffolds
[24]	0.05	–	Chondrocyte	Increase	Increase	Not measured	No scaffolds
[25]	0.5	10–20%	Chondrocyte	Increase	Increase	Increase	Fibrin-polyurethane

**Table 6:** Experimental fluid shear by different bioreactor and scaffolds and effects on chondrocyte proliferation and matrix composition

	RPM	Scaffold	Cell proliferation	Collagen	GAG	Proteoglycan	Type for bioreactor
[26]	80	PGA	Chondrocyte	Increase 80%	Increase	Not measured	Spinner flask
[27]	50	PGA	Chondrocyte	Increase	Increase	Increase	Spinner flask
[28]	50	No scaffolds	Chondrocyte	Increase 125%	Increase 60%	Not measured	Spinner flask
[29]	90	osteochondral tissue	Chondrocyte	Increase	Increase	Increase	Spinner bioreactor
[30]	50–140	No scaffolds	No cell	Not measured	Not measured	Not measured	Wavy-walled bioreactor
[31]	–	chitosan/gelatin	Adipose-derived stem cells	Increase	Increase	Increase	Spinner flask
[32]	–	No scaffolds	Chondrocyte	Not measured	Not measured	Not measured	3D finite element model
[33]	–	No scaffolds	No cell	Not measured	Not measured	Not measured	Hollow fiber (mathematical modeling)
[24]	–	No scaffolds	Chondroprogenitor cells	Increase	Increase	Increase	Model
[34]	–	No scaffolds	No cell	Not measured	Not measured	Not measured	Hollow fiber (mathematical modeling)

**Table 7:** Effect of perfusion bioreactor on chondrocyte proliferation and matrix composition

	Pa	Rate	Cell proliferation	Collagen	GAG	Proteoglycan	Scaffold
[35]	–	0.33 ml/min	Chondrocyte	Collagen2 increase 240%	300% (S)180% (NS)	Increase 35% aggrecan	Collagen sponges
[36]	–	1 μm/s	Chondrocyte	155% increase	Increase 184%	Increase 118%	PLLA/PGA
[37]	0.01	–	Chondrocyte	Increase	Increase	Increase	Micro-porous scaffolds
[38]	0.01	0.5 ml/min	Chondrocyte	Increase	Increase	Increase	Polyestherurethane foams
[39]	0.1.	2 ml/min	Chondrocyte	Increase	Increase	Increase	Explant
[40]	–	0.1 ml/min	Human mesenchymal stem cells	Increase	Increase	Increase	Polycaprolactone (PCL) beads
[41]	–	3 ml/min	Chondrocyte	Increase	Increase	Increase	Alginate
[42]	–	0.33 ml/min	Chondrocyte	–	Increase	Increase	Electrospun poly(ε-caprolactone
[43]	–	1000, 300 μm/s	Chondrocyte	Increase	Increase	Increase	Collagen sponges
[44]	0.05–0.45	0.005–0.045 ml/min	Chondrocyte	Increase	Increase	Increase	Polyurethane
[45]	–	10 μm/s	Chondrocyte	Increase	Increase	Increase	No scaffolds

**Table 8:** Effect of perfusion bioreactor with low shear on chondrocyte proliferation and matrix composition

	Pa	RPM	Rate	Cell proliferation	Collagen	GAG	Proteoglycan	Scaffold	Bioreactor
[46–49]	1.10	–	0.5–2	Chondrocyte	Increase	Increase	Increase	Alginate	Perfusion
[50]	–	15–30	–	Chondrocyte	Increase	Increase	Increase	Hyaluronan benzyl ester non-woven	Rotating
[51]	–	–	–	No cell	Increase 33%	Increase 68%	Not measured	No scaffolds	Rotating
[52]	–	–	–	Chondrocyte	Increase 39%	increase 95%	–	No scaffolds	-
[53]	17 kPa	1.32 ml h^−1^		Chondrocyte	Increase	Increase	Increase	Scaffold-free	acoustofluidic perfusion

#### Contact shear

During the physiological situation, cartilage is rubbing against either cartilage or produce contact shear. Several studies attempted to stimulate the solid-on-solid, contact shear using bioreactors and different scaffold (Table 5). The results of Waldman et al. study demonstrated that intermittent application of dynamic shearing forces (2% shear strain amplitude at a frequency of 1 Hz) increased both collagen and proteoglycan synthesis and improves the quality of cartilaginous tissue [15]. Also, in the study of Frank et al. through metabolic studies and application of sinusoidal macroscopic shear deformation (rotational resolution is 0.0005°), increase in the synthesis of proteoglycan and proteins was detected [16]. Several studies examined the tissue shear loading (0.01–1.0 Hz, using 1–3% sinusoidal shear strain amplitudes) on chondrocyte biosynthesis and revealed that the synthesis of protein by approximately 50% and proteoglycans by approximately 25% increased [17, 18]. Colombo t al. developed and validated a multi-axial device named RPETS with sinusoidal motion frequency between 0.05 and 0.5 Hz [19]. Also, Di Federico et al. described an in vitro mechanical system to chondrocyte-seeded agarose constructs (compressive and shear loading regimen at 1 Hz for up to 48 h) to investigate the response of chondrocytes to a complex physiologically relevant deformation profile [20]. In the study of Chai et al., bovine articular chondrocytes were seeded in 2% agarose gels subjected to a 24-h dynamic compression regime (1 Hz, 2.5% dynamic strain amplitude, 7% static offset strain) that increased proteoglycan synthesis and total glycosaminoglycans (GAG) accumulation [21]. In a similar study, Fitzgerald et al. subjected intact cartilage explants to 1–24 h of continuous dynamic compression or dynamic shear loading at 0.1 Hz. Results showed that most matrix proteins were upregulated by 24 h of dynamic compression or dynamic shear [22]. Malaeb et al. built a four-chamber bioreactor to apply hydrostatic pressure, compression, shear, and torsion (frequency of 0.5 Hz). Results showed that the system was capable of delivering a variety of mechanical stimuli in native cartilage [23]. In a study, Juhasz et al. investigated the loading scheme (0.05 Hz, 600 Pa; for 30 min) on chondroprogenitor cells of 4-day-old chicken embryos. The results showed that several cartilage matrix constituents, including collagen type II and aggrecan core protein, as well as matrix-producing hyaluronan synthases increased [24]. Also, Vainieri et al. developed a model of osteochondral defect from bovine stifle joints using bioreactor that mimics the multi-axial motion of an articulating joint. Results revealed that proteoglycan 4 and cartilage oligomeric matrix protein, mRNA ratios of collagen type II to type I, and aggrecan to versican were markedly improved [25].

#### Fluid shear

Fluid flow bioreactor development is consistent with the hypothesis that increase in nutrient and wastes transfer lead to increase in cell metabolism. Several bioreactors including spinner flask and wavy-walled bioreactor developed for the purpose (Table 6).

Gooch et al. investigated the effects of the hydrodynamic environment by using spinner flask (80 RPM) on bovine calf chondrocytes seeded on polyglycolic acid meshes. The finding of the study was higher fractions of collagen and more GAG in chondrocytes [26]. Also, Bueno et al. developed a wavy-walled bioreactor to provide high-axial mixing environment to the cultivation of cartilage constructs. Polyglycolic acid scaffolds seeded with bovine articular chondrocytes and resulted increased cell proliferation and extracellular matrix deposition [27]

Vunjak-Novakovic et al. investigated the effect of bovine articular chondrocytes seeded in fibrous polyglycolic acid in well-mixed spinner flasks. This environment resulted in the formation of 20–32-micron diameter cell aggregates that enhanced the kinetics of cell attachment [28]. In a similar study, Theodoropoulos et al. placed articular cartilage of bovine metacarpal-phalangeal joints in spinner bioreactors and maintained on a magnetic stir plate at 90 rotations per minute (RPM). The study found that there was a significant increase in collagen content, the expression of membrane type 1 matrix metalloproteinase (MT1-MMP), and aggrecan [29]. Study of Bilgen et al. that applied a wavy-walled bioreactor (WWB) demonstrated the importance of characterization of mixing and impact of changes in bioreactor geometry and operating conditions [30]. In the study of Song et al. in a spinner flask, adipose-derived stem cells (ADSCs) seeded with chitosan/gelatin hybrid hydrogel scaffolds. ADSCs differentiated into chondrocytes and expressed more proteoglycans and cell distribution [31]. In another study, Cortez et al. developed a 3D finite element model to mechanical simulate (5%, 10%, and 15% of compressive strain with frequencies of 0.5 Hz, 1 Hz, and 2 Hz) the diffusion and transport of nutrients. The findings showed that fluid shear stress improved the solute transport and chondrocyte activity [32]. Also, Chapman et al. applied a model to predict the optimal flow rate of culture medium into the fiber lumen [33]. Juhasz et al. in a study investigated the loading scheme (0.05 Hz, 600 Pa; for 30 min) on chondroprogenitor cells and showed an increase in cartilage matrix constituents of chicken embryos, including collagen type II and aggrecan core protein, as well as matrix-producing hyaluronan synthases [24]. Also, Pearson et al. applied a model of fluid flow, nutrient transport, and cell distribution using a hollow fiber membrane bioreactor. With the model and the effect of mechanotransduction on the distribution investigated [34].

#### Perfusion bioreactor

Transfer nutrient through the three-dimensional biomaterial and tissue constructs is one of the serious problem and limitations of fluid shear bioreactors. Therefore, direct perfusion bioreactor with different flow rates investigated and developed to overcome the nutrient limitations (Table 7). Mizuno et al. in a study cultured bovine articular chondrocytes in 3D collagen sponges with medium perfusion (0.33 mL/min) for up to 15 days. Interestingly, the results demonstrated that these conditions that are beneficial for other cell types inhibit chondrogenesis by articular chondrocytes [35]. Pazzano et al. cultured chondrocytes seeded on PLLA/PGA under to 1 μm/s flow and demonstrated a 118% increase in DNA content, a 184% increase in GAG content, and a 155% increase in hydroxyproline content [36]. Also, culture of bovine articular chondrocytes seeded on micro-porous scaffolds under a median shear stress of 1.2 and 6.7 mPa, promoted the formation of extra-cellular matrix specific to hyaline cartilage [37]. In another study, bovine articular chondrocytes seeded on polyesterurethane foams and cultured for 2 weeks under flow rate (0.5 ml/min). The results of study indicated that mean content in DNA and GAG increased [38]. In a similar study, the culture of human chondrocytes in bioreactor applied loading (0.1 MPa for 2 h) and perfusion (2 ml) led to increase of COL2A1 expression and decrease of COL1A1 and MMP-13 expression [39]. Carmona-Moran and Wick applied perfusion bioreactor to promote chondrogenesis of human mesenchymal stem cells. Results of this culture condition showed that after day 14, collagen deposition and proteoglycan deposition increased [40]. Yu et al. developed the tubular perfusion system (TPS) and cultured chondrocytes encapsulated in alginate for 14 days and demonstrated that 3 mL/min does not damage the chondrocytes. This culture condition resulted in increased gene expression levels of aggrecan, type II collagen, and superficial zone protein [41]. In a similar study, Dahlin et al. cultured chondrocytes seeded onto electrospun poly(ε-caprolactone) under perfusion condition and demonstrated an increase in chondrocyte proliferation and glycosaminoglycan production [42]. Mayer et al. cultured human articular chondrocytes seeded in collagen sponges with a bidirectional perfusion bioreactor. Results indicated that perfusion bioreactor and cocktail of soluble factors, the BIT (BMP-2, insulin, thyroxin) improved the distribution and quality of cartilaginous matrix [43]. Raimondi et al. investigated the effects of three different perfusion flow rates and shear stress levels (0.005, 0.023 ml/min and 0.045 ml/min) to chondrocytes detachment from cellularized constructs. Results indicated the number of detached cells increased [44]. Also, the finding of Tonnarelli et al. study indicated that culture of chondrocytes bioreactor culture conditions support chondrogenic differentiation [45].

#### Low shear bioreactor

Low shear mixing improves the growth of cells on three-dimensional scaffolds and applies minimal loading to constructs. Rotating bioreactors are the most popular devices to apply low shear mixing (Table 8). Several studies investigated the effect of flow-induced shear stress by perfusion bioreactor on alginate encapsulating chondrocytes. Tissue construct subjected to shear showed morphological features, which are characteristic of natural cartilage [46–49]. Also, Tognana et al. examined the culture of bovine calf chondrocytes and hyaluronan benzyl ester non-woven mesh under perfusion bioreactor. Results indicated that this culture condition improved chondrogenesis and integrative repair in engineered cartilage [50]. In a similar study, Tsao et al. developed a mathematical model to characterize cell-medium interactions and demonstrated that experimental results support the numerical simulation [51]. The finding of Martin et al.’s study indicated that composition and mechanical properties of engineered cartilage (highest fractions of glycosaminoglycans and collagen) can be modulated by the culture conditions [52]. Li et al developed acoustofluidic perfusion bioreactors to overcome the limitations of conventional static cartilage bioengineering [53].

## Conclusion

In the field of tissue engineering, several bioreactors developed at once and at different times to apply mechanical forces to cartilage constructs. Mechanism of shear stress on chondrocyte, briefly, can be hypothesized as the following [24, 29, 35, 41]:

1. Altered expression of aggrecan and collagen type II
2. Altered cartilage oligomeric matrix protein (COMP) serum levels, consequently, organizing the arrangement binding of glycosaminoglycans, integrins, and collagen
3. Induction of apoptosis signals
4. Altered expression of integrin.
